# Inhibition of ERK1/2 down-regulates the Hippo/YAP signaling pathway in human NSCLC cells

**DOI:** 10.18632/oncotarget.2974

**Published:** 2015-01-23

**Authors:** Bin You, Yi-Lin Yang, Zhidong Xu, Yuyuan Dai, Shu Liu, Jian-Hua Mao, Osamu Tetsu, Hui Li, David M. Jablons, Liang You

**Affiliations:** ^1^ Department of Surgery, Helen Diller Family Comprehensive Cancer Center, University of California, San Francisco, CA, USA; ^2^ Department of Thoracic Surgery, Beijing Chao-Yang Hospital, Affiliated with Capital University of Medical Science, Beijing, People's Republic of China; ^3^ Life Sciences Division, Lawrence Berkeley National Laboratory, University of California, Berkeley, CA, USA; ^4^ Department of Otolaryngology–Head and Neck Surgery, University of California, San Francisco, CA, USA

**Keywords:** non-small cell lung cancer, extracellular signal regulated kinases, Hippo pathway, yes-associated protein, inhibition

## Abstract

Alterations of the EGFR/ERK and Hippo/YAP pathway have been found in non-small cell lung cancer (NSCLC). Herein, we show that ERK1 and ERK2 have an effect on the Hippo/YAP pathway in human NSCLC cells. Firstly, inhibition of ERK1/2 by siRNA or small-molecular inhibitors decreased the YAP protein level, the reporter activity of the Hippo pathway, and the mRNA levels of the Hippo downstream genes, *CTGF*, *Gli2*, and *BIRC5*. Secondly, degradation of YAP protein was accelerated after ERK1/2 depletion in NSCLC cell lines, in which YAP mRNA level was not decreased. Thirdly, forced over-expression of the ERK2 gene rescued the YAP protein level and Hippo reporter activity after siRNA knockdown targeting 3′UTR of the ERK2 gene in NSCLC cells. Fourthly, depletion of ERK1/2 reduced the migration and invasion of NSCLC cells. Combined depletion of ERK1/2 had a greater effect on cell migration than depletion of either one separately. Finally, the MEK1/2 inhibitor Trametinib decreased YAP protein level and transcriptional activity of the Hippo pathway in NSCLC cell lines. Our results suggest that ERK1/2 inhibition participates in reducing YAP protein level, which in turn down-regulates expression of the downstream genes of the Hippo pathway to suppress migration and invasion of NSCLC cells.

## INTRODUCTION

Non-small cell lung cancer (NSCLC), a common malignancy, has a known association with mitogen-activated protein kinase (MAPK) signal transduction pathways [1–3]. Extracellular signal regulated kinases (ERK1/2) are important components of the MAPK signal pathway, which is mediated by Epidermal Growth Factor Receptor (EGFR). ERK1/2 proteins, after activation through the Ras-Raf-MEK kinase cascade, can translocate into the nucleus and activate various effectors, including transcriptional factors, kinases, and phosphatases, to promote cell differentiation and proliferation [4]. Amplification of genes that encode ERK and overexpression of ERK have been found in human NSCLC [5, 6]. In clinical and pre-clinical studies, disrupting the MAPK/ERK pathway in cancer cells through inhibiting Raf or MEK often induced negative feedback or paradoxical activation of ERK and resulted in drug resistance [7–9]. Direct inhibition of ERK1/2 is becoming a new strategy in cancer treatment [10–12].

Another cancer pathway identified in NSCLC is the Hippo (also known as the Salvador-Warts-Hippo) pathway [13–15]. In normal cells, Yes-associated protein (YAP), one of the major mediators, is negatively regulated by upstream components of the Hippo pathway [16, 17]. The Hippo/YAP pathway has shown a correlation with stem cell renewal and differentiation, a crucial step of oncogenic transformation [18]. YAP plays a key role in regulating organ size and cancer development [19, 20], and as a growth promoter, YAP is found at elevated levels in many human cancers [21, 22]. Moreover, YAP up-regulation was identified in NSCLC patient tissues [13, 22].

Hippo is an evolutionarily conserved pathway in cells, and just a few oncogenic mutations have been discovered in human cancers to date [23]. Understanding what causes alteration of the Hippo pathway in cancer cells could lead to new therapeutic strategies. On the other hand, as a central transducer of many biological signals, ERK1/2 mediates a series of molecular alterations and has complex crosstalk with other signaling pathways [24–26]. We sought to investigate whether ERK1/2 expression is associated with Hippo pathway activity by analyzing the effects of ERK1/2 inhibition on the Hippo pathway activity in human NSCLC cells. We analyzed the transcriptional activity of Hippo downstream genes and YAP protein expression after ERK1/2 knockdown by using small interfering RNA (siRNA) or small-molecular inhibitors. We report for the first time that ERK1/2 inhibition contributes to down-regulation of the Hippo pathway in human NSCLC cells and suppressed migration and invasion of the cell.

## RESULTS

### Inhibition of ERK1/2 down-regulates YAP protein expression

**Figure 1:**
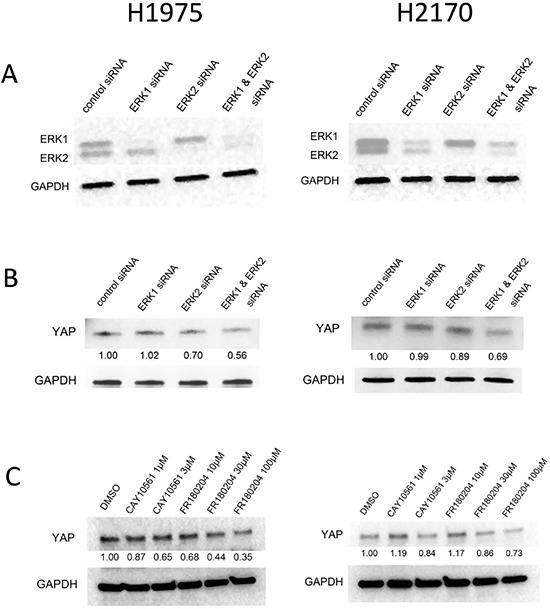
Western blotting analysis of YAP protein level after ERK1/2 inhibition in NSCLC cells **(A)** Decreased ERK1 and ERK2 protein level after siRNA treatment in H1975 and H2170 cells. **(B)** Decreased YAP protein level after ERK1/2 inhibition by siRNAs (100 nM) in H1975 and H2170 cells. **(C)** Dose-dependent decrease of YAP protein level in cells treated with ERK inhibitors.

To investigate whether inhibition of ERK1/2 influences YAP, we examined the protein level of YAP after ERK1/2 knockdown. The efficiency of ERK inhibition after 48 hours of siRNA treatment in NSCLC H1975 and H2170 cells was assessed by western blotting (Figure 1A). We found that YAP protein level decreased after ERK1/2 knockdown by siRNA (Figure 1B). To verify the suppression of YAP protein by ERK inhibition, we analyzed YAP protein level in the NSCLC cells after treatment with small-molecular inhibitors. We used two ERK inhibitors: CAY10561 (N-[1-(3-chloro-4-fluorophenyl)-2-hydroxyethyl]-4-[4-(3-chlorophenyl)-1-Hpyrazol-3-yl]-1H-pyrrole-2-carboxamide), a highly selective inhibitor of ERK2 [27], and FR180204 (5-(2-Phenyl-pyrazolo [1, 5-a] pyridin-3-yl)-1H-pyrazolo [3, 4-c] pyridazin-3-ylamine), a selective inhibitor of ERK1/2 [28]. The results showed that YAP expression decreased in a dose-dependent manner in both cell lines treated with either CAY10561 or FR180204, in contrast to what occurred after control treatment with DMSO (Figure 1C). These findings suggest that ERK1/2 inhibition decreases the protein level of YAP in NSCLC cells.

### ERK1/2 Inhibition does not decrease the mRNA level of YAP

**Figure 2:**
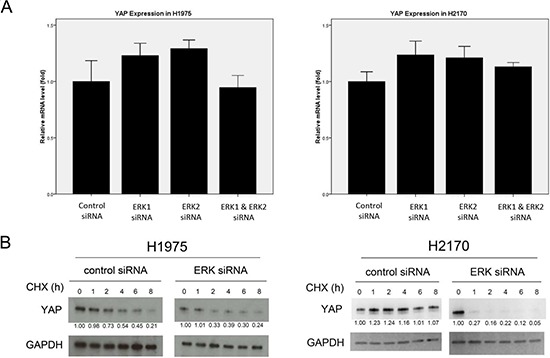
Analysis of YAP expression after ERK1/2 inhibition in NSCLC cells **(A)** YAP mRNA level in H1975 and H2170 cells after ERK inhibition by siRNA was measured using RT-PCR. **(B)** Degradation of YAP protein was analyzed in H1975 and H2170 cells after ERK1/2 inhibition by siRNA.

We next tested whether ERK1/2 inhibition decreases the mRNA level of YAP in NSCLC cells. The mRNA level of YAP after ERK1/2 siRNA treatment in H1975 and H2170 cells was analyzed by using semi-quantitative real-time PCR (RT-PCR). We found that YAP mRNA levels in the cells treated with ERK1/2 siRNA did not differ significantly from those treated with non-targeting control siRNA (Figure 2A; Suppl. Table S1). These results suggest that ERK1/2 inhibition down-regulates YAP at the protein level rather than at the mRNA level.

### Depletion of ERK1/2 promotes YAP degradation

To investigate whether the decrease of YAP protein level mediated by ERK1/2 inhibition is through degradation, we examined the half-life of YAP protein in H1975 and H2170 cells after ERK inhibition. After ERK1/2 inhibition by siRNA, cells were treated with protein inhibitor cycloheximide and YAP protein level was analyzed in the cells at 0, 1, 2, 4, 6 and 8 hours by western blotting (Figure 2B). In H1975 cells, the half-life of YAP protein is about 6 hours after treatment of cycloheximide and non-targeting siRNA control. After ERK1/2 inhibition by siRNA, YAP protein reached a low level at 2 hours after cycloheximide treatment, suggesting that the half-life of YAP after ERK1/2 depletion is about 2 hours in H1975 cells. In H2170 cells treating with non-targeting siRNA, the YAP protein level showed only minor decrease within 8 hours. After ERK1/2 depletion, the YAP protein level decreased dramatically at 1 hour after cycloheximide treatment, suggesting a half-life of less than 1 hour in H2170 cells. These results showed that ERK1/2 depletion decreased the half-life of YAP in both NSCLC cell lines, suggesting ERK1/2 depletion promotes YAP degradation.

### ERK1/2 inhibition down-regulates hippo reporter activity and downstream gene expression

**Figure 3:**
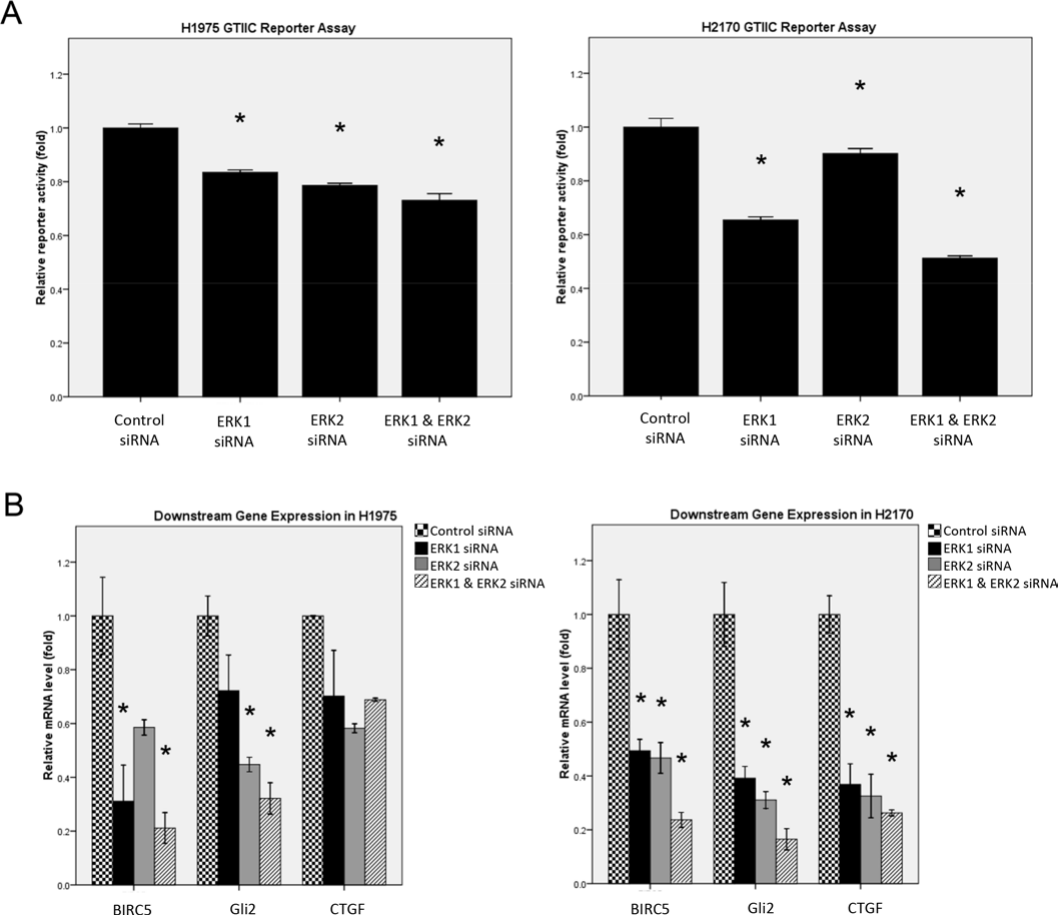
Analysis of Hippo pathway activity after ERK1/2 inhibition by siRNA in NSCLC cells **(A)** GTIIC reporter activity of Hippo pathway after ERK1/2 inhibition by siRNA was analyzed in H1975 and H2170 cells (*P < 0.05, one-way ANOVA and Scheffe multiple comparisons). **(B)** Decreased expression of BIRC5, Gli2, and CTGF, the downstream genes of Hippo pathway, after ERK1/2 inhibition by siRNA in H1975 and H2170 cells (*P < 0.05, One-way ANOVA and Scheffe multiple comparisons).

As YAP is a central mediator of the Hippo pathway, we next investigated whether ERK1/2 suppression affects the reporter activity of the Hippo pathway in NSCLC cells. GTIIC reporter activity of Hippo pathway was analyzed in H1975 and H2170 cells after ERK inhibition by siRNA (Figure 3A). In H1975 cells, the reporter activity decreased by 16.5% after ERK1 silencing, 21.4% after ERK2 silencing, and 26.9% after ERK1/2 silencing. In H2170 cells, the reporter activity decreased by 34.5% after ERK1 silencing, 9.8% after ERK2 silencing, and 48.8% after ERK1/2 silencing. Our results show that ERK1/2 inhibition by siRNA significantly decreased GTIIC reporter activity of the Hippo pathway in both H1975 and H2170 cells (*P* < 0.05) compared to that of their respective control cells treated with non-targeting siRNA. Moreover, the mRNA level of the Hippo pathway downstream genes *CTGF*, *Gli2*, and *BIRC5* analyzed in these cells was consistently down-regulated after ERK1/2 depletion (Figure 3B, Suppl. Table S2). Furthermore, Hippo reporter activity and Hippo downstream gene transcription decreased more after depletion of both ERK1 and ERK2 than after depletion of ERK1 or ERK2 alone.

**Figure 4:**
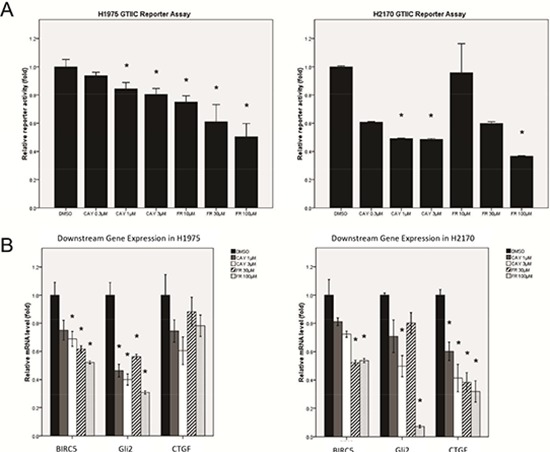
Analysis of Hippo pathway activity after ERK1/2 inhibition by small molecule inhibitors in NSCLC cells **(A)** A dose-dependent decrease in GTIIC reporter activity of the Hippo pathway after ERK inhibition by ERK inhibitors (CAY10561 or FR180204) in H1975 and H2170 cells (*P < 0.05, One-way ANOVA and Scheffe multiple comparisons). **(B)** A dose-dependent decrease in mRNA level of BIRC5, CTGF, and Gli2, the downstream genes of Hippo pathway, after ERK inhibition by ERK inhibitors (CAY10561 and FR180204) in H1975 and H2170 cells (*P < 0.05, One-way ANOVA, Scheffe multiple comparisons).

We further examined the effect of ERK inhibition on Hippo pathway activities using the small-molecular ERK2 inhibitor CAY10561 and the ERK1/2 inhibitor FR180204. After treatment with either inhibitor, Hippo reporter activity decreased in a dose-dependent manner in both H1975 and H2170 cells, as compared to the DMSO control (*P* < 0.05) (Figure 4A). Quantitative RT-PCR analysis also showed a dose-dependent decrease of *CTGF*, *Gli2* and *BIRC5* transcription in both cell lines (*P* < 0.05) (Figure 4B, Suppl. Table S3). Together, these results suggest that ERK1/2 inhibition down-regulates the reporter activity and downstream gene transcription of the Hippo pathway in NSCLC cells.

### Forced over-expression of the ERK2 gene rescues hippo/YAP expression during ERK2 depletion

**Figure 5:**
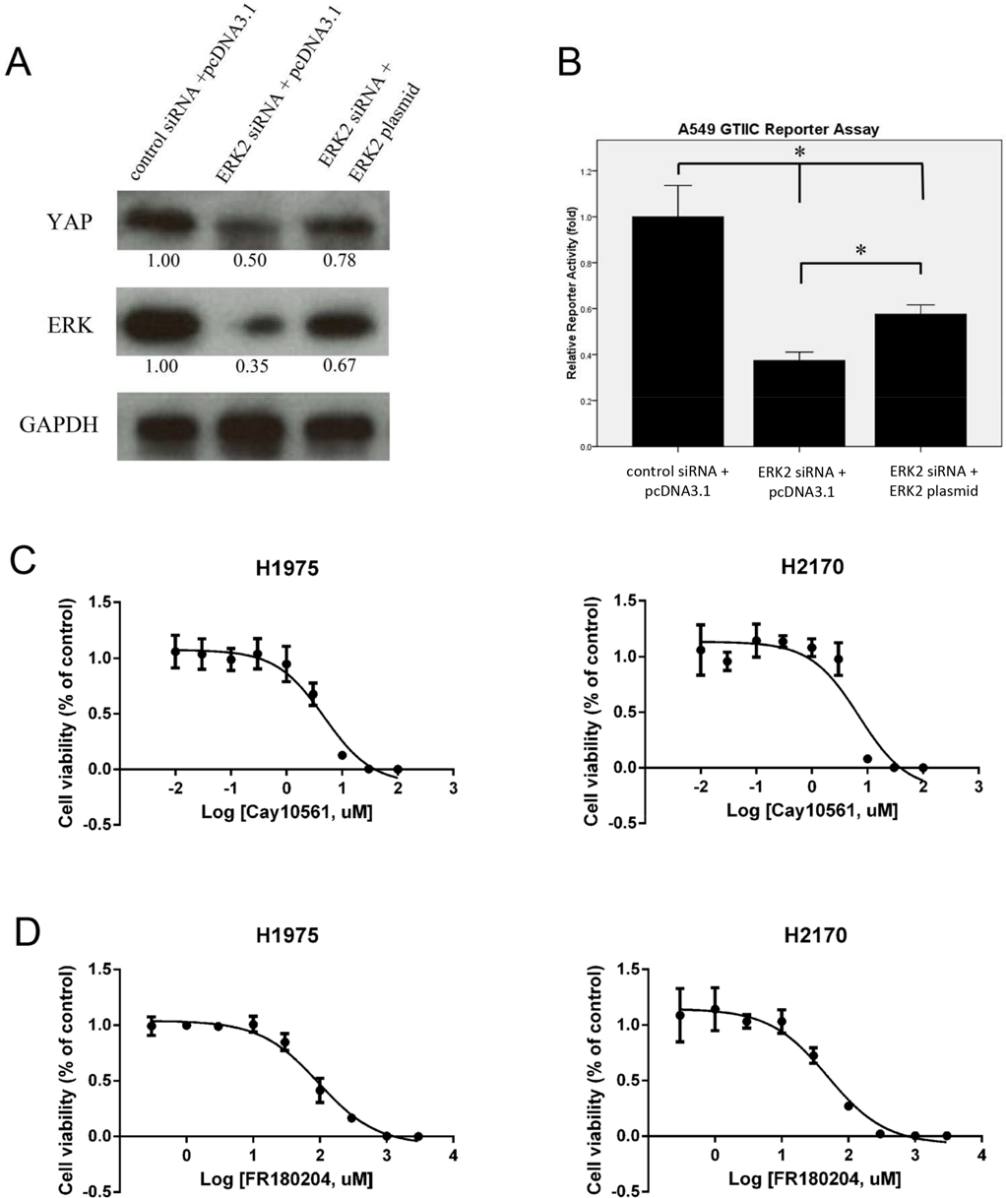
Expression of YAP/Hippo pathway and cell viability analysis after ERK inhibition in NSCLC cells **(A)** Western blotting analysis of YAP, ERK, and GAPDH after ERK2 silencing by siRNA and/or forced over-expression of the ERK2 gene in A549 cells. **(B)** GTIIC reporter activity of the Hippo pathway after ERK2 silencing by siRNA and/or forced over-expression ERK2 gene in A549 cells (*P < 0.05. One-way ANOVA and Scheffe multiple comparisons). **(C)** Cell viability analysis in H1975 and H2170 cells after ERK inhibitor CAY10561 treatment. **(D)** Cell viability analysis in H1975 and H2170 cells after ERK inhibitor FR180204 treatment.

To verify that YAP protein expression can be regulated by ERK expression, we analyzed YAP protein level after ERK2 inhibition and/or forced over-expression of the ERK2 gene in NSCLC cell line A549. For this, we used the ERK2 siRNA, which targeted the 3′UTR end of the ERK2 gene. We found that YAP protein level decreased after ERK2 depletion in A549 cells (Figure 5A), results that were similar to what we found after ERK inhibition using a pooled ERK2 siRNA. After forced overexpression of the ERK2 gene, YAP protein level was 50% increase compared to that in the cells treated with ERK2 3′UTR siRNA only (Figure 5B). After 3′UTR siRNA treatment, Hippo reporter activity was significantly reduced by 62.6%, compared to that in the cells treated with control non-targeting siRNA (*P* < 0.05), and Hippo reporter activity was rescued by more than 30% after forced overexpression of the ERK2 gene in cells (*P* < 0.05). Together, these results suggest that Hippo/YAP expression is regulated by ERK expression in NSCLC cells.

### ERK inhibitors suppress viability of NSCLC cells

We next tested the effects of ERK inhibitors on the viability of NSCLC cells. H1975 and H2170 cells were treated with ERK inhibitors CA10561 and FR180204 at different doses for 48 hours. Cell viability was assayed and IC50 of each cell line was calculated based on the dose-response curves (Figure 5C, 5D). IC50 of CAY10561 was 4.74 μM in H1975 cells and 7.01 μM in H2170 cells. IC50 of FR180204 in was 95.36 μM in H1975 cells and 49.0 μM in H2170 cells. These results show that ERK inhibition suppressed cell viability in a dose-dependent manner in both NSCLC cell lines.

### ERK1/2 inhibition restrains migration and invasion of NSCLC cells

**Figure 6:**
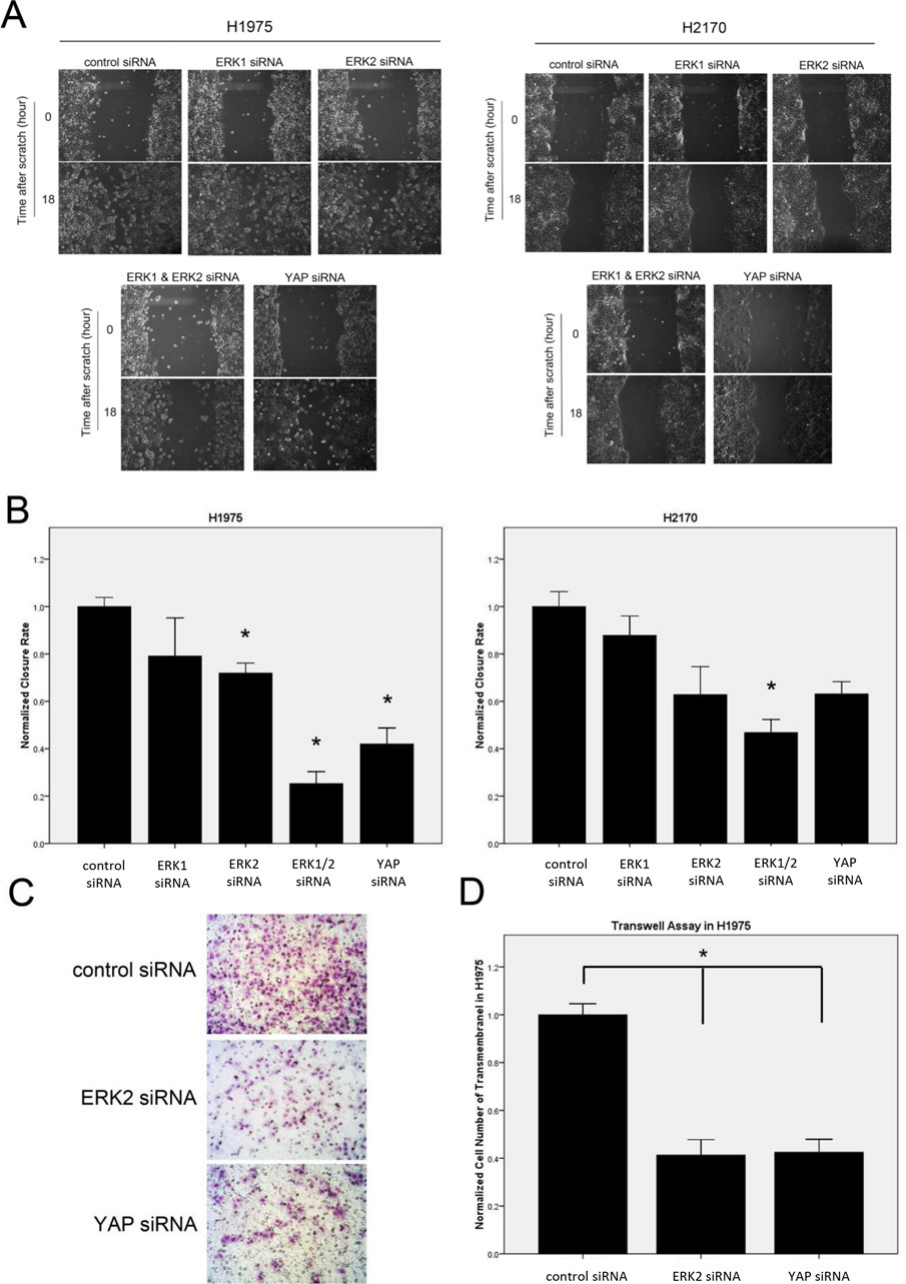
Analysis of cell migration and invasion abilities after ERK inhibition in NSCLC cells **(A)** Decrease in cell migration ability after ERK or YAP inhibition by siRNA in H1975 and H2170 cells. **(B)** Quantitative analysis of migration assay result (*P < 0.05, one-way ANOVA, Scheffe multiple comparisons). **(C)** Decrease in cell invasion ability in H1975 cells after ERK2 or YAP silencing by siRNA. Images were taken under a 20 × objective lens. **(D)** Quantitative analysis of the number of cells that invaded the lower side of the membrane in each experimental group (*P < 0.05, One-way ANOVA and Scheffe multiple comparisons).

To assess the effect of ERK1/2 inhibition on the migration ability of NSCLC cells, we carried out a wound-healing assay using H1975 and H2170 cells. Cells transfected with ERK1/2 siRNA or YAP siRNA for 48 hours were scratched with a 200 μl pipette tip, and the rate of wound closure was observed for 18 hours, when cells in the control group were proximally confluent. In both cell lines, wound closure rates were significantly decreased after ERK1/2 depletion compared to that in the control group (Figure 6A, 6B; *P* < 0.05). Depletion of YAP yielded observations similar to those after depletion of both ERK1 and ERK2, suggesting that ERK1/2 inhibition restrains the migratory ability of the NSCLC tumor cells possibly through YAP down-regulation. Moreover, depletion of both ERK1 and ERK2 resulted in more significant inhibition of tumor cell migration than depletion of either alone (*P* < 0.05). This greater effect of ERK1/2 combined is consistent with our findings in the mRNA expression and reporter assay of Hippo pathway.

To test the effect of ERK inhibition on the invasion ability of NSCLC cells, a transwell assay was performed using H1975 cells after siRNA treatment for 48 hours. The number of the cells that invaded the lower side of the membrane decreased significantly 20 hours after ERK2 siRNA or YAP siRNA treatment, compared to that in the control group (Figure 6C, 6D; *P* < 0.05). Similarly, the invasive ability of NSCLC cells was decreased when either ERK2 or YAP was depleted, suggesting that ERK2 inhibition suppresses the invasive ability of the NSCLC tumor cells possibly through the Hippo/YAP pathway.

### MEK1/2 inhibition causes down-regulation of the hippo/YAP pathway

**Figure 7:**
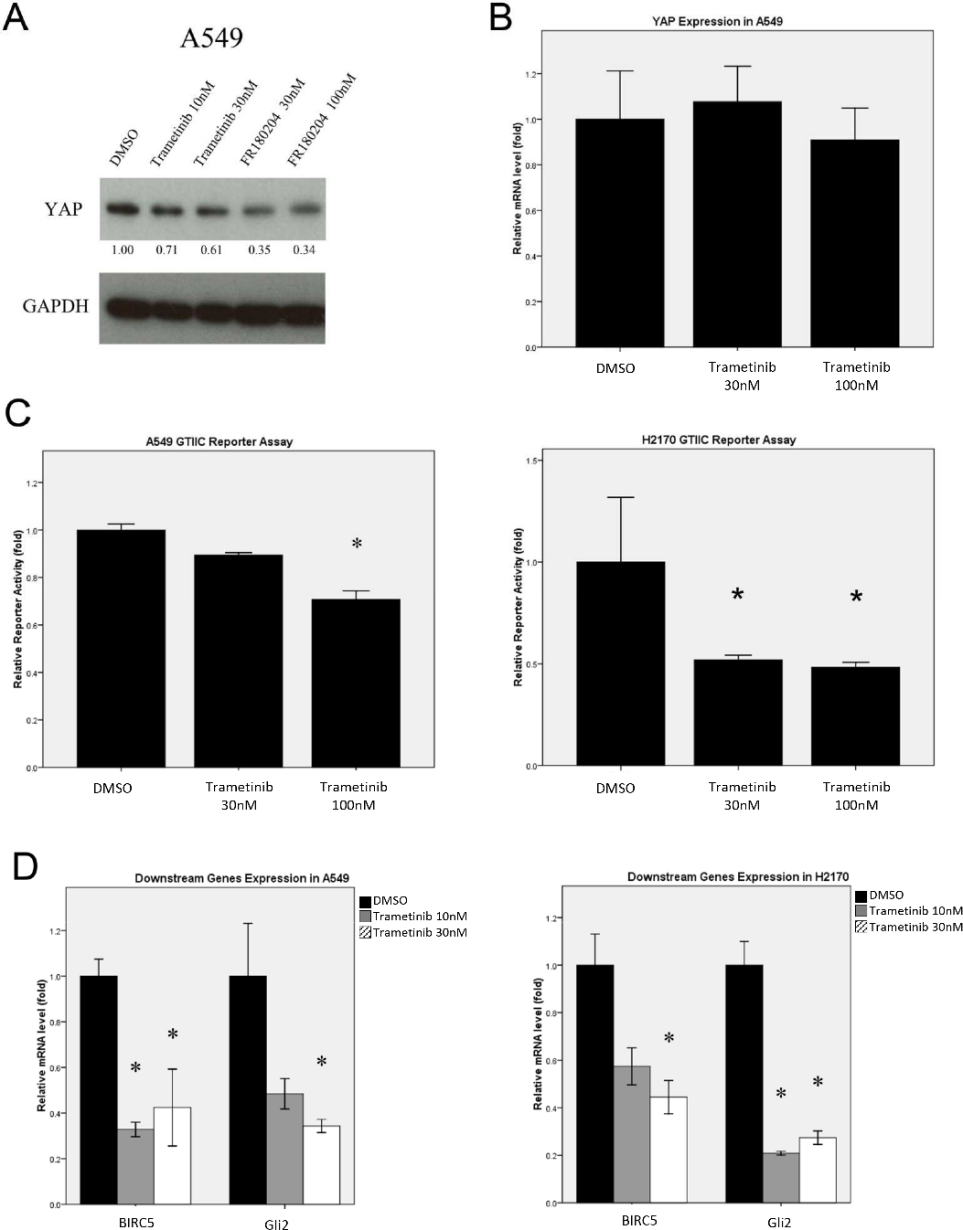
Analysis of Hippo/YAP pathway activity after MEK1/2 inhibition in NSCLC cells **(A)** Western blotting analysis of YAP expression in A549 cells treated with MEK1/2 inhibitor Trametinib or ERK inhibitor FR180204. **(B)** The mRNA level of YAP in A549 cells treated with MEK1/2 inhibitor Trametinib was measured using RT-PCR. **(C)** A dose-dependent decrease in GTIIC reporter activity of the Hippo pathway was analyzed in A549 and H2170 cells treated with MEK1/2 inhibitor Trametinib (*P < 0.05, One-way ANOVA and Scheffe multiple comparisons). **(D)** Decreased mRNA levels of BIRC5 and Gli2, the downstream genes of the Hippo pathway, in A549 and H2170 cells treated with MEK1/2 inhibitor Trametinib (*P < 0.05, One-way ANOVA, Scheffe multiple comparisons).

To investigate whether inhibiting MEK, an upstream regulator of ERK signaling, affects Hippo pathway activity, we analyzed YAP expression in NSCLC A549 cells treated with the MEK1/2 inhibitor Trametinib (N-(3-(3-cyclopropyl-5-(2-fluoro-4-iodophenylamino)-6, 8-dimethyl-2, 4, 7-trioxo-3, 4, 6, 7-tetrahydropyrido [4,3-d] pyrimidin-1(2H)-yl) phenyl) acetamide, GSK1120212 [29] or the ERK inhibitor FR180204. We found that YAP protein level decreased after treatment with either inhibitor, in contrast with what occurred in cells treated with DMSO (Figure 7A). The mRNA level of YAP in A549 cells after Trametinib treatment was analyzed using RT-PCR, and there was no significant difference in YAP mRNA level in the cells with Trametinib or DMSO treatment (Figure 7B, Suppl. Table S4). Together, these results suggest that MEK inhibition decreased YAP protein level, but has only a minimal effect on YAP transcription in the cells. Furthermore, we examined the reporter activity and downstream gene expression of the Hippo pathway in NSCLC cells after MEK inhibition. GTIIC reporter activity of the Hippo pathway was decreased in A549 and H2170 cells after treatment with the MEK inhibitor Trametinib, compared to that of their respective control cells (Figure 7C, *P* < 0.05). Similarly, the mRNA levels of Hippo downstream genes, *Gli2* and *BIRC5*, were consistently down-regulated by MEK inhibition in A549 cells (Figure 7D, Suppl. Table S5, *P* < 0.05). The effect of MEK inhibition was similar to that of ERK inhibition on Hippo/YAP pathway activity. Our results suggest that MEK inhibition mediated YAP down-regulation at the protein level and thereby suppresses the reporter activity and downstream gene expression of the Hippo pathway in NSCLC cell lines.

## DISCUSSION

The results of our study suggest that ERK1 and ERK2 have previously unrecognized effects on the Hippo pathway in human NSCLC cells. Several lines of evidence support this relationship between ERK1/2 and the Hippo/YAP pathway. First, in NSCLC cell lines, ERK1/2 inhibition by siRNA or small-molecular inhibitors down-regulates the protein level of YAP, and in turn suppresses transcriptional activity of the Hippo pathway. Additionally, these effects of ERK inhibition can be rescued by forced over-expression of ERK2 in the cells. Second, ERK1/2 inhibition reduces the viability, migration, and invasion of NSCLC cells. Third, ERK1/2 knockdown decreased YAP expression at the protein level, but not the mRNA level, and the results of a protein degradation assay indicated that ERK1/2 inhibition down-regulates YAP protein level, at least partially, through promoting its degradation.

The correlation between ERK and the Hippo pathway, which have similar effects on cell properties and oncogenesis, has been studied. Mitogen-activated protein kinase 1 (MEK1) was recently reported to be closely correlated with YAP in human liver cancer cells, but ERK was deemed irrelevant to the Hippo/YAP pathway [30]. We analyzed the association of ERK and YAP expression in a lung cancer tissue array using immunohistochemistry. Our results show that YAP and ERK expression were significantly associated in 78 lung adenocarcinoma tumors (Suppl. Figure S1A–B; *P* < 0.05). In addition, the results of our cell experiments suggest that ERK1/2 inhibition may negatively regulate the Hippo/YAP pathway in human NSCLC cells. Moreover, we performed a YAP rescue experiment after ERK2 knockdown by a 3′UTR siRNA. The result shows that the viability of NSCLC cells was rescued by forced overexpression of the YAP gene (Suppl. Figure S2; *P* < 0.05), suggesting that the suppressive effect of ERK inhibition on cellular biological behavior may be partly through down-regulating activity of the Hippo/YAP pathway. As the Hippo pathway is known to be a regulator of cancer stem cells [18, 31–33] and is involved in cell adhesion and migration, which are closely associated with cancer progression and metastasis [34–36], our results suggest that ERK1/2 should be a therapeutic target in this process.

Regulation of ERK expression is important in cancer cells [37, 38], and inhibitors of MEK, a kinase that activates ERK, have been studied in clinical trials. Our study shows that the MEK inhibitor Trametinib down-regulated the Hippo/YAP pathway in a way that was similar to what occurred after ERK inhibition. However, inhibiting upstream components of the MAPK pathway, such as Raf or MEK, often induces negative feedback or paradoxical activation of ERK and results in drug resistance [7–9]. These findings, together with our results, suggest that the ERK inhibitor should be further evaluated for its therapeutic potential [10, 11, 39]. ERK inhibitors have already entered clinical trials for cancer treatment, for example, MK-8353/SCH900353, a clinical grade analogue of SCH772984, BVD-523, and RG7842 [40].

Targeting the Hippo pathway is a new direction for cancer drug development [41]. In human NSCLC, the Hippo pathway and some of its downstream genes, such as *CTGF*, *Gli2* and *BIRC5*, are associated with the occurrence and development of the disease [42–45]. The results of our study show that in NSCLC cells, the expression of these Hippo downstream genes can be reduced by ERK1/2 inhibition and also suggest that the stability of YAP protein could be reduced by ERK1/2 inhibition. Therefore, blocking the Hippo pathway by ERK1/2 inhibition may be a therapeutic strategy in cancer treatment.

The mechanism by which ERK regulates YAP stability is not clear. In the Hippo pathway, YAP is negatively regulated by a core cassette consisted of LATS1, LATS2, MST1, and MST2 kinases [17, 23, 46]. In human NSCLC, LATS1 expression has been shown to be associated with YAP expression [47], and MST1 overexpression enhances YAP phosphorylation at the Ser127 site in human NSCLC cells [48]. Moreover, the Hippo pathway can be negatively regulated by epidermal growth factor (EGF) signaling [49], in which ERK functions as one of the vital effectors (Suppl. Figure S3). EGF signaling has been shown to dissociate the Hippo core complex, resulting in LATS inactivation, YAP dephosphorylation at Ser127, YAP nuclear accumulation, and CTGF transcriptional activation [50]. We detected nuclear staining of YAP in human NSCLC samples using immunohistochemistry (Suppl. Figure S1C), suggesting that EGF signaling is activated and regulates the Hippo pathway through inhibiting Hippo kinase(s) in the tumor cells. Other studies seeking to understand the regulation of Hippo pathway have shown that MAPK can regulate Ajuba family LIM domain protein (Jub), Wilms tumor protein 1-interacting protein (WTIP), or KIBRA, which are all regulators of the Hippo pathway [51, 52]. Raf-1 was also shown to regulate MST2 and the Hippo pathway [53]. Taken together, the results from our study and others lead us to hypothesize that ERK regulates YAP stability through the Hippo pathway in NSCLC cells. Further studies are warranted to test this hypothesis and to understand how MAPK regulates the Hippo pathway in human NSCLC.

In our study, the migration of cancer cells was inhibited more after ERK2 silencing than after ERK1 silencing, and depletion of both together showed a greater effect on Hippo pathway activity and cell migration than either of them did separately. These results suggest that inhibition of both ERK1 and ERK2 may be involved in regulating Hippo pathway activities. To date, research about ERK inhibitors has mainly focused on ERK2 [54], but collective inhibition of ERK1 and ERK2 may offer a better approach to therapy for human NSCLC.

In summary, we report that ERK1/2 inhibition down-regulates the protein level of YAP via promoting its degradation, and thereby reduces expression of the downstream genes of the Hippo pathway. This regulation of YAP may impair migratory and invasive activity of tumor cells in human NSCLC. Our results provide interesting insights about crosstalk between the two cell signaling pathways.

## METHODS

### Cell culture

Human NSCLC cell lines H1975, H2170 and A549 were obtained from American Type Culture Collections (Manassas, VA). Cell lines were maintained in RPMI-1640 supplemented with 10% heat-inactivated fetal bovine serum, penicillin (100 mg/ml) and streptomycin (100 mg/ml), and were cultured at 37°C in a humid incubator with 5% CO_2_.

### Small molecules, siRNA, plasmid DNA, and antibodies

The SMARTPool siRNA targeting p42 MAPK (ERK2), p44 MAPK (ERK1), and YAP were purchased from Thermo Scientific Dharmacon (Pittsburgh, PA). Non-targeting siRNA was used as control (Thermo Scientific Dharmacon, Pittsburgh, PA). Additional ERK2 siRNA (Hss163792) targeting the 3′UTR end of ERK2 gene purchased from Life Technologies (Grand Island, NY) was used in the rescue studies. The ERK2 inhibitor CAY10561 (CAS 933786–58–4) was obtained from Cayman Chemical Company (Ann Arbor, Michigan), the ERK1/2 inhibitor FR180204 (3706) from Tocris Bioscience (San Diego, CA), and the MEK inhibitor Trametinib from Selleckchem (Houston, TX). The pUSE ERK2 plasmid DNA used to over-express the ERK2 gene in the cells was purchased from Millipore. The pBaby-YAP plasmid DNA used to over-express the YAP gene in the cells was purchased from Addgene. Antibodies for ERK and YAP used in this study were purchased from Cell Signaling, Inc. (Danvers, MA). The HRP-linked secondary antibodies were from GE Healthcare Bio-Sciences Corp (Piscataway, NJ).

### Small molecule treatment and siRNA transfection

Cells were plated in 24-well plates (for PCR or reporter assay) or 6-well plates (for western blot or wound-healing assay) 24 hours before treatment. Cells were transfected with 100 nmol/L of siRNA using Lipofectamine RNAiMAX (Invitrogen, Carlsbad, CA) according to the manufacturer's protocol. After transfection for 48 hours, cells were harvested for further analysis. Small molecule inhibitors CAY10561, FR180204 and Trametinib were dissolved in DMSO. Cells treated with small molecule inhibitors at a series of dosages were grown for 24 hours before being harvested.

### RNA isolation, cDNA synthesis and quantitative real-time RT-PCR

Total RNA was extracted from cells using the RNeasy Mini kit (Qiagen, Valencia, CA). The cDNA was transcribed from 500 ng of total RNA using iScript cDNA Synthesis Kits (Bio-Rad, Hercules, CA), according to the manufacturer's protocol. The cDNA was used as the template for real-time PCR detection using TaqMan Technology on an Applied Biosystems 7000 sequence detection system (Applied Biosystems, Foster City, CA). Expression of *BIRC5*, *CTGF*, *Gli2* genes and endogenous control gene b-glucuronidase (*GUSB*) were detected using the primer and probe sequences commercially available (Applied Biosystems) and analyzed using Relative Quantification Software (Applied Biosystems).

### Luciferase reporter assay

The 8 × GTIIC-luciferase plasmid (Addgene, Cambridge, MA) and Renilla luciferase pRL-TK plasmid (Promega, Madison, WI) were co-transfected into cell lines. The transfection reagents were Lipofectamine RNAiMAX or Lipofectamine 2000 (Invitrogen, Carlsbad, CA), depending on treatment with siRNA or small molecule inhibitors. After 48 hours, cells were harvested and transferred into a 96-well plate for analysis by using the Dual-Luciferase Reporter Assay Kit (Promega, Madison, WI). Detection of luminescent signaling was performed on a GloMax-96 Microplate Luminometer (Promega, Madison, WI) according to the manufacturer's instructions.

### Western blot analysis

Total protein was extracted from cell lines using M-PER Mammalian Protein Extraction Reagent (Thermo) supplied with Complete Protease Inhibitor Cocktails (Roche, Lewes, UK), according to manufacturers' protocols. The protein concentrations were measured with the Pierce BCA Protein Assay Kit (Thermo). A total of 10 μg of proteins were run on 4~20% gradient SDS–polyacrylamide gels (Bio-Rad Laboratories, Inc., Hercules, CA) and transferred to Immobilon-P nitrocellulose membranes (Millipore, Bellerica, MA). The membranes were blocked in 5% nonfat milk and then probed with the primary antibodies overnight at 4°C. The membranes were incubated with appropriate secondary antibodies, followed by detection using an ECL blotting analysis system (Amersham Pharmacia Biotech, Piscataway, NJ).

### Protein degradation assay

The ERK1/2 siRNA and control siRNA in a concentration of 100 nM were transfected into the NSCLC cells. After 48 hours of transfection, the cells were treated with 100 μg/ml cycloheximide (Sigma), the inhibitor of protein synthesis, and harvested at the time points of 0, 1, 2, 4, 6 and 8 hours. Total proteins were extracted and expression of YAP was analyzed by western blot.

### Cell viability assay

Cells were cultured in a 96-well plate and treated with different doses of ERK inhibitors (CA10561: 0, 0.01, 0.03, 0.1, 0.3, 1, 3, 10, 30, 100 μM; FR180204: 0, 0.3, 1, 3, 10, 30, 100, 300, 1000, 3000 μM). After 48 hours of incubation, cells were lysed and luminescent signaling was generated by a CellTiter-Glo Luminescent Cell Viability Assay reagent (Promega). Luminescent signaling was measured on the GloMax-96 Microplate Luminometer. Proportional cell viability was analyzed with GraphPad Prism6 software (GraphPad Software, Inc., La Jolla, CA), which was used to calculate dose-response curves and IC50.

### Wound-healing assay

Sub-confluent cultures of cell lines were transfected with ERK1, ERK2, ERK1/2, YAP or control siRNAs. The plates were scratched by a 200 μl pipette tip at 48 hours after transfection. The fresh medium was replaced and cells were grown continuously. Phase contrast images were taken with a Primo Vert microscope (ZEISS, Gottingen, Germany) at the time of the scratch (0 h) and every 6 hours after. Scratch areas were quantified using ImageJ software. Wound-closure rates were calculated as percentages of the initial distance (0 h), and normalized using the data from control siRNA.

### Transwell invasion assay

The transwell invasion assay was performed in a 6-well plate transwell system (Corning Incorporated, USA). The transwell inserts were coated with 100 μl matrigel and incubated at 37°C for 2 hours. H1975 cells were transfected with 100 nM control siRNA, ERK2 siRNA or YAP siRNA for 48 hours. After siRNA treatment, cells were harvested and resuspended in serum-free medium. The cells were seeded on the upper chamber of the transwell, and the lower chamber was infused with 2 ml complete growth medium (10% FBS). The transwell was incubated at 37°C for 20 hours, and then the gel and cells in the upper chamber were wiped out. After methanol fixation, the membrane was stained by hematoxylin for 40 seconds. Phase contrast images were taken and the cells on the lower side of the membrane were counted in six random visual fields under a 20 × objective lens.

### Tissue samples and immunohistochemistry (IHC)

Fresh lung tumor tissues were obtained from patients who were undergoing surgical resection of the primary tumor. All human tissue samples were obtained and analyzed in accordance with procedures approved by the institutional review board of the University of California, San Francisco (IRB H8714–22 942–01). The tissue microarray sections were immunostained as previously described [55]. The following scoring system was used: −, no stain; +, weak staining (10% or above stained cellularity considered as positive); ++, moderate staining (30% or above stained cellularity considered as positive); +++, strong staining (50% or above stained cellularity considered as positive). All scoring was done under low power objective lens (20 ×) with a Zeiss Axioscop 2 microscope (Carl Zeiss Inc, Germany). Images were taken under 20 × or 40 × objective lens.

### Statistical analysis

Data are expressed as mean ± standard deviation (SD) from three independent experiments. All statistical analyses were performed using the SPSS 17.0 for Windows software system (SPSS Inc, Chicago, IL). One-way ANOVA followed by Scheffe multiple comparisons were used to compare the differences among multiple groups. A significant difference was considered when the *P* value from a two-tailed test was < 0.05.

## SUPPLEMENTARY FIGURES AND TABLES


