# Supplementary material for: Inhibition of ERK1/2 down-regulates the Hippo/YAP signaling pathway in human NSCLC cells
Source: Oncotarget. 2015 Jan 23;6(6):4357–68. doi: 10.18632/oncotarget.2974 (PMC4414195; doi:10.18632/oncotarget.2974)
Supplement: Supplementary file 1 [file oncotarget-06-4357-s001.pdf]

SUPPLEMENTARY FIGURES AND TABLES

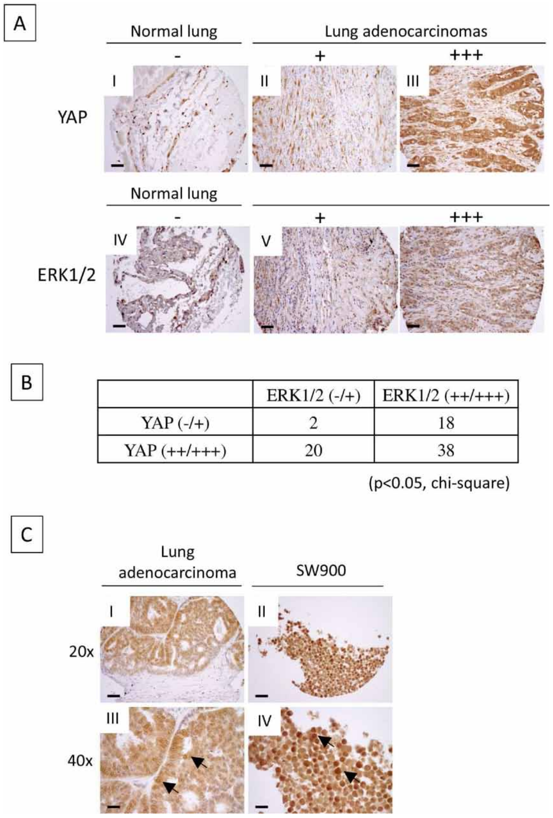

	ERK1/2 (-/+)	ERK1/2 (++/+++)
YAP (-/+)	2	18
YAP (++/+++)	20	38

Supplementary Figure 1: Analysis of YAP and ERK1/2 protein expression in clinical lung adenocarcinoma tissue using immunohistochemistry (IHC). **(A)** YAP and ERK1/2 expression were analyzed in identical tissue sections from (I, IV) normal lung or (II–VI) lung adenocarcinomas. Images were taken under 20 × objective lens. (Scale bar: 180 μm) **(B)** YAP and ERK1/2 expression were significantly associated in 78 lung adenocarcinoma tumors ($p < 0.05$, chi-square). **(C)** Staining of YAP was localized in nuclei (arrows) of lung adenocarcinomas and NSCLC cell line SW900 under 20 × or 40 × objective lens. (Scale bar: 180 μm).

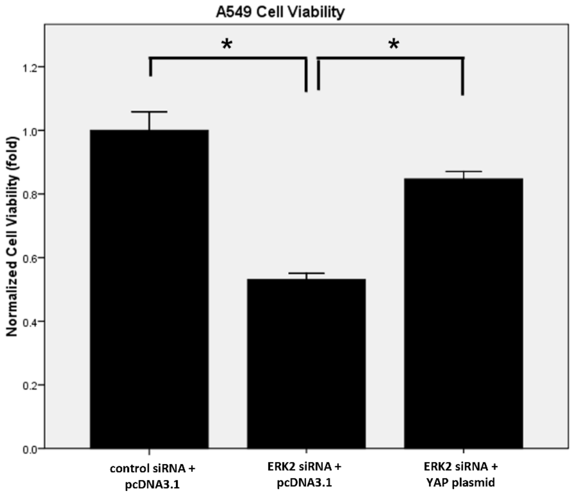

Supplementary Figure 2: Cell viability analysis of A549 cells after ERK2 silencing and/or forced over-expression of the ERK2 gene. Cell viability was significantly decreased after ERK2 silencing by siRNA, compared to the control cells (*P < 0.05). Cell viability was significantly increased after ERK2 silencing and forced over-expression of the ERK2 gene in A549 cells, compared to that with ERK2 silencing alone (*P < 0.05, One-way ANOVA and Scheffe multiple comparisons).

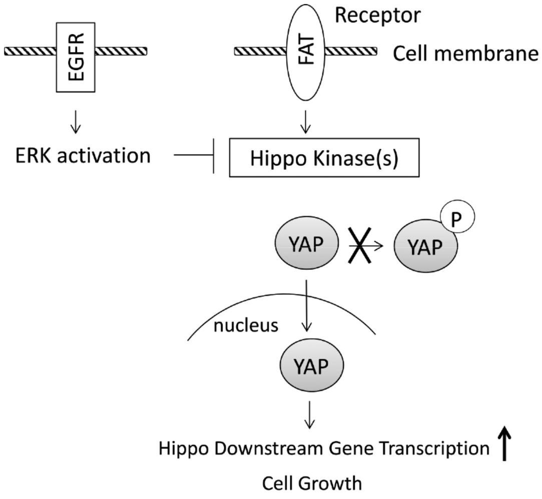

Supplementary Figure 3: Model of the Hippo pathway regulated by EGF signaling. Activated Hippo signaling turns on Hippo kinase activity. Activated EGF signaling turns on ERK activation, which inhibits Hippo kinase activity. Without Hippo kinase activity, YAP enters the cell nucleus instead of being phosphorylated, and promotes Hippo downstream gene transcription and cell growth.

Supplementary Table S1: The mRNA Level of YAP after ERK1/2 Depletion Detected by RT-PCR in Two NSCLC Cell Lines

	H1975	H2170
siERK1	123.1%	123.7%
	P = 0.247	*P* = 0.080
siERK2	129.2%	121.1%
	P = 0.116	*P* = 0.125
siERK1/2	94.6%	113.2%
	P = 0.963	*P* = 0.435

The data for each group is shown as the percentage of that of control siRNA treatment. The treated groups did not differ significantly from the control group. One-way ANOVA followed by Scheffe multiple comparisons.

Supplementary Table S2: Decrease in Downstream Gene Expression after ERK1/2 Knockdown by RT-PCR in Two NSCLC Cell Lines

	H1975			H2170		
	BIRC5	CTGF	Gli2	BIRC5	CTGF	Gli2
siERK1	68.9%	29.8%	27.8%	50.6%	63.1%	60.8%
	P = 0.025*	*P* = 0.253	*P* = 0.209	*P* = 0.010*	*P* = 0.001*	*P* = 0.002*
siERK2	41.5%	41.8%	55.2%	53.3%	67.5%	68.9%
	P = 0.116	*P* = 0.108	*P* = 0.010*	*P* = 0.007*	*P* = 0.001*	*P* = 0.001*
siERK1/2	78.9%	31.2%	67.8%	76.3%	73.7%	83.5%
	P = 0.013*	*P* = 0.230	*P* = 0.003*	*P* = 0.001*	*P* < 0.001*	*P* < 0.001*

Decrease in downstream gene expression is shown as the percentage of that of control siRNA treatment. *$P < 0.05$, one-way ANOVA followed by Scheffe multiple comparisons.

Supplementary Table S3: Decrease in Downstream Gene Expression after ERK Inhibition by RT-PCR in Two NSCLC Cell Lines

	H1975			H2170		
	BIRC5	Gli2	CTGF	BIRC5	Gli2	CTGF
CAY10561 1 µM	25.0%	53.6%	25.5%	18.8%	29.2%	39.8%
	$P = 0.124$	$P < 0.001$*	$P = 0.577$	$P = 0.246$	$P = 0.189$	$P = 0.038$*
CAY10561 3 µM	31.1%	60.0%	39.5%	27.6%	50.2%	58.6%
	$P = 0.044$*	$P < 0.001$*	$P = 0.204$	$P = 0.050$	$P = 0.014$*	$P = 0.003$*
FR180204 30 µM	38.4%	43.8%	11.8%	47.7%	19.8%	61.6%
	$P = 0.013$*	$P = 0.002$*	$P = 0.954$	$P = 0.001$*	$P = 0.507$	$P = 0.002$*
FR180204 100 µM	47.8%	69.1%	21.8%	46.3%	92.9%	68.0%
	$P = 0.003$*	$P < 0.001$*	$P = 0.704$	$P = 0.002$*	$P < 0.001$*	$P = 0.001$*

Decrease in downstream gene expression is shown by percentage of the data of DMSO treatment. (*$P < 0.05$), one-way ANOVA followed by Scheffe multiple comparisons.

Supplementary Table S4: mRNA Level of YAP after MEK Inhibition Detected by RT-PCR in A549 Cells

	A549
Trametinib 30 nM	107.7%
	$P = 0.951$
Trametinib 100 nM	91.0%
	$P = 0.934$

The data for each group is shown as the percentage of that of DMSO treatment. The treated groups did not differ significantly from the control group. One-way ANOVA followed by Scheffe multiple comparisons.

Supplementary Table S5: Decrease in Downstream Gene Expression after MEK Inhibition by RT-PCR in Two NSCLC Cell Lines

	A549		H2170	
	BIRC5	Gli2	BIRC5	Gli2
Trametinib 10 nM	32.8%	48.4%	57.4%	20.9%
	$P = 0.013$*	$P = 0.103$	$P = 0.056$	$P < 0.001$*
Trametinib 30 nM	42.4%	34.3%	44.5%	27.4%
	$P = 0.026$*	$P = 0.044$*	$P = 0.019$*	$P < 0.001$*

Decrease in downstream gene expression was shown by percentage of the data of DMSO treatment. (*$P < 0.05$), one-way ANOVA followed by Scheffe multiple comparisons.
